# Medical Students' Corner: Lessons From COVID-19 in Equity, Adaptability, and Community for the Future of Medical Education

**DOI:** 10.2196/23604

**Published:** 2020-10-09

**Authors:** Simran Mann, Shonnelly Novintan, Yasmin Hazemi-Jebelli, Daniel Faehndrich

**Affiliations:** 1 School of Medicine Imperial College London London United Kingdom

**Keywords:** medical education, COVID-19, student equity, community, adaptability, medical student

## Abstract

As UK medical students, we recently completed 3 months of remote learning due to the COVID-19 pandemic, before taking online end-of-the-year exams. We are now entering our final year of medical school. Based on our experiences and our understanding of others’ experiences, we believe that three key lessons have been universal for medical students around the world. The lessons learned throughout this process address the need for a fair system for medical students, the importance of adaptability in all aspects of medical education, and the value of a strong medical school community. These lessons can be applied in the years to come to improve medical education as we know it.

## Introduction

Medical education and future careers are sometimes colloquially referred to as a conveyor belt: they involve a set pathway with a known structure of assessments at each key step, and as a medical student, your focus is on continuously moving forward. While medicine itself is unpredictable by nature, medical education follows a familiar structure. This is why the impact of COVID-19 has been so explosive. For the first time, our placements, our annual exams, and our subsequent progression through the medical degree have all been canceled, delayed, or altered significantly.

As UK medical students, we recently completed 3 months of remote learning, before sitting end-of-the-year exams online, and we are now entering our final year of medical school. Our remote learning mostly consisted of online lectures delivered by clinicians from various hospital sites. Based on our experiences and our understanding of others’ experiences, we believe that three key lessons have been universal for medical students around the world.

## Equity: A Fair System for All Students

In recent months, medical schools have had to provide remote teaching that is accessible and appropriate for all students. This has included a range of media outlets for online lectures, tutorials, and virtual clinics [1-4]. However, this shift to online learning has uncovered an “equity gap” within medical schools, as it has become apparent that many students lack access to adequate technology or working space.

In many medical schools, including our own, all teaching has been conducted remotely, and online assessments have replaced the traditional “exam hall” assessment [5]. Executing this has highlighted the disparity in access to devices, reliable Wi-Fi, and suitable working spaces at home. For example, some students are not equipped with a quiet space at home or a reliable device with a webcam and microphone, limiting their ability to partake in videoconferencing. Furthermore, many students have inadequate Wi-Fi, which makes video calls slow or “glitchy” and impedes learning.

Unfortunately, the impact of the pandemic on medical education has been far harsher on low-income students, and universities have scrambled to mitigate this. In our medical school, students regularly use institution-provided mobile devices [6], which has reduced the inequity between students during remote learning, but many would argue that they still desperately need a physical library space and campus Wi-Fi. Our campus libraries have been closed in response to the pandemic, which has been problematic for students with limited working space at home.

We must also address the fact that these adversities already affected year-round studying—even before medical schools felt the impact of the COVID-19 pandemic. A campus library with a computer is vital for a student from a low-income background, as there is often no alternative working space at home. At our medical school, the educational experience of students who rely on library facilities is already disproportionately affected by factors such as restricted library opening hours, limited availability of computers, or insufficient working space. Thus, as campuses are reopening, universities should re-evaluate the accessibility of studying facilities throughout the academic year.

The pandemic may open a wider conversation about resource availability for medical students. Following the disruption to regular teaching, many students bought access to compensatory resources such as textbooks, flashcards, and educational websites. Learning is streamlined by comprehensive, “all-in-one” resources in which content is updated alongside emerging evidence and changing guidelines. Here, individual-guided learning during COVID-19 has confirmed the academic advantages of wealth: if you can afford these subscriptions, you can mitigate adverse circumstances and optimize your remote learning.

Addressing this deep-rooted inequity is difficult but important. Perhaps universities should strive to keep their “free” resources as updated and comprehensive as privately available resources. The strain of the pandemic has also been greater in students who rely on part-time jobs; thus, COVID-19 has reminded universities of the importance of financial safeguards for working students. This strain can be minimized through means-tested bursaries, which reduce the need for part-time jobs and may also help students to afford valuable, private learning resources.

Ultimately, the obvious inequities in assessment and education must not be treated as a discrete issue but should be addressed outside of the context of COVID-19. The pandemic has illustrated the usefulness of technology for remote learning, but now we risk overlooking the need for physical working spaces. Campus libraries must be kept open throughout the year, with social distancing measures as necessary, to accommodate students with no working space or reliable Wi-Fi at home. Furthermore, universities must strive to mitigate inequalities between students, with fully accessible academic resources and means-tested bursaries.

The first lesson from medical education in the times of COVID-19 is a need for a fair system. Hopefully, the renewed focus on equal resources for learning and assessments will prompt us to tackle the insidious causes of inequity amongst medical students.

## Adaptability: Our Responsiveness in Unprecedented Times

COVID-19 has required us to rapidly re-evaluate how we learn and assess within medical education. This has relied on the adaptability of individuals, resources, and the assessments themselves.

For years, many medical schools, including our own, have been recording lectures and making them available for students online—a style of teaching that has been proven popular and effective since it allows students to learn at their own pace and in their own time [7,8]. As remote teaching has become the norm, students in medical schools such as ours can adapt more readily, as we are already familiar with the platforms and format of recorded lectures. In the context of canceled lectures, it is also easy to make online banks of prerecorded lectures accessible for students who are learning remotely.

Similarly, many teaching hospitals already implement “virtual multidisciplinary teams” (MDTs) over video conferencing software [9,10]. The existing infrastructure has made it easier for these institutions to adapt their teaching by having medical students attend MDTs virtually. The same principle of an adaptable infrastructure can be applied to the role of virtual clinics [11,12] in medical education. History-taking skills can be practiced with simulated patients or even real patients, as more health care providers begin to use video and phone consultations.

While we can shift lectures, MDTs, and some clinics into a remote format, our ability to develop clinical skills has suffered. Canceled placements and reduced patient contact have made it harder to practice physical exams and clinical procedures. This was a necessary step to minimize risks of COVID-19 transmission, but now we risk inadequate training for this generation of future doctors. History-taking can be practiced during online, small-group teaching, but the practicalities of an abdominal examination or peripheral venous cannulation are more difficult to simulate.

Some virtual tools have been used, with varying results. Barriers to using virtual reality and computer-generated patients include access to technology, organizational culture, and real-life applicability of the simulated environment [13-15]. The fact that many medical schools do not routinely use these tools has limited our ability to quickly implement them as a substitute for clinical practice. However, as these tools become more necessary to medical education, they can be refined and incorporated into regular use. Our medical school has never used such tools, but the faculty is beginning to trial “virtual ward rounds” and may use them regularly in the future.

A key step in improving our adaptability will involve the integration of virtual tools into our medical school curricula. This can ensure an adequate standard of clinical education, even in situations with minimal patient contact.

## Community: Peer-to-Peer Support

A wide range of virtual platforms have been used by faculty and clinicians to support remote learning for medical students. Personally, however, we have found that much of our adaptation to virtual learning has been centered around peer-to-peer support.

Close interaction between teachers and students is fundamental in medical education. To understand a condition, students must discuss and question each step in the patient journey, and this is facilitated by individual-level teaching. Usually, we experience this during placements as doctors discuss specific cases with us and encourage us to take histories or examine patients. Small-group teaching can be substituted for this, but to provide a regular program of remote, small-group teaching for students year-round would require pre-existing infrastructure. Our faculty has succeeded in providing remote lessons for large groups, but small-group teaching has not been rolled out.

After placements were suspended, we struggled to find a substitute for the discussions that we normally experience during a placement. Therefore, peer-to-peer teaching has guided our adaptation to remote learning.

Our medical school community has responded to COVID-19 with a strong sense of unity. Within extracurricular clubs and social groups, many students have independently divided their curricula into topics and led informal group tutorials using video conferencing software. Furthermore, many senior students ran informal, virtual tutorials for students in earlier years on content that we have previously covered and have been examined on. Students also benefited from similar informal tutorials run by alumni who have studied the course and are now practicing clinicians. Thus, our networks throughout medical school have facilitated peer-to-peer support across different stages of medical education.

The opportunity to invest a significant amount of time into student-led tutorials has been pivotal in our education this year. We confirmed that students’ understanding is improved when taught by somebody at a close stage in their medical career, who instinctively pitches content at our level of knowledge [16]. Furthermore, teaching and being questioned by our peers consolidate our own knowledge. Finally, our teaching skills have developed through feedback from peers.

Each tutorial was organized within a student-run, nonacademic club or society. The nature of student societies is ideal for virtual learning, as small groups form naturally, and friendships facilitate relaxed discussion. There are obvious limitations to students attempting to fulfil professional teaching roles. However, in these circumstances, peer-to-peer teaching has been a safety net for many of us, preventing us from falling behind in our learning. We believe that student-led learning could be better supported by all medical schools, including our own, by encouraging tutorial groups and perhaps providing suggested tutorial frameworks for students who have never taken on a teaching role before.

The pandemic has broadened our understanding of the value of a strong medical school community. Having a peer network is fundamental for coping with the emotional demands of medicine, particularly in the context of COVID-19 [17,18]. Regarding academic demands, we have learned that students will support each other in unprecedented circumstances, when it is needed most.

## Conclusion

The COVID-19 pandemic has forced us to re-evaluate key aspects of medical education. The lessons learned throughout this process address the need for a fair system for medical students, the importance of adaptability in all aspects of medical education, and the value of a strong medical school community. These lessons can be applied in the years to come to improve medical education as we know it.

## Abbreviations

MDT: virtual multidisciplinary team
